# *Alistipes finegoldii* in Blood Cultures from Colon Cancer Patients

**DOI:** 10.3201/eid1308.060662

**Published:** 2007-08

**Authors:** Lukas Fenner, Véronique Roux, Pascal Ananian, Didier Raoult

**Affiliations:** * Hôpital de la Timone, Marseille, France; † Hôpital de la Conception, Marseille, France; 1 Current affiliation: University Hospital Basel, Basel, Switzerland

**Keywords:** *Alistipes finegoldii*, blood culture, colon cancer, abdominal surgery, letter

**To the Editor:** *Alistipes finegoldii* was previously isolated from appendiceal tissue samples in children with acute appendicitis and from perirectal and brain abscess material (*1*,*2*). 16S rDNA sequencing studies showed that this bacterium clustered with *A. putredinis* (Figure) in the Bacteroidetes group (*4*). We describe the first cases, to our knowledge, of bacteremia due to *A. finegoldii* in 2 patients with colon cancer who underwent surgical resection.

**Figure:**
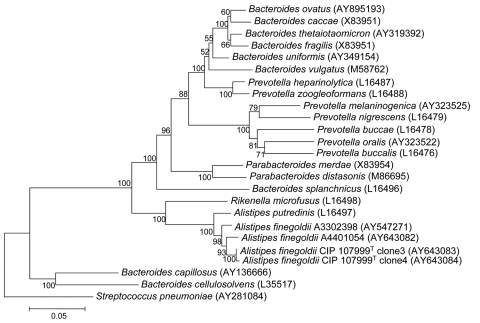
Phylogenetic tree inferred from comparison of the 16S rRNA gene sequences of genera *Bacteroides*, *Parabacteroidetes*, *Prevotella,* and *Alistipes*. Nucleotide accession numbers for the sequences used to construct this dendrogram are given in parentheses. The tree was constructed with MEGA version 2.1 (www.megasoftware.net). Distance matrices were determined following the assumptions described by Kimura (*3*) and were used to elaborate the dendrogram with the neighbor-joining method. Bar, 0.05-nt change per nucleotide position. *Streptococcus pneumoniae* was used as the outgroup.

The first patient was a 61-year-old woman with colorectal carcinoma and liver metastasis, who underwent chemotherapy consisting of 6 cycles of oxaliplatin (the FOLFOX scheme, a chemotherapy regimen consisting of fluorouracil [5 FU], folinic acid, and oxaliplatin). In September 2003, a left colectomy, resection of metastasis in the left side of the liver, and a ligation of the right portal vein were performed. Two months later, in a second step, a right hepatectomy was done. On postoperative day 5, the patient had a fever up to 39.8°C and leukocyte count of 8.49 g/L (68% polymorphonuclear leukocytes). Two blood cultures were performed before antimicrobial drug therapy based on amoxicillin/clavulanic acid and amikacin was started. After receiving this therapy, the patient recovered rapidly. One of the 2 anaerobic blood cultures was positive. Gram-negative bacilli were isolated (strain 3302398). Antimicrobial susceptibility testing showed decreased susceptibility to vancomycin, cefotetan, and penicillin G. The strain produced β-lactamase as determined by Cefinase test (Becton Dickinson, Le Pont de Claix, France).

The second patient was a 64-year-old man with colon cancer who was receiving palliative chemotherapy (16th cycle, FOLFOX scheme); he was seen in March 2004 with a fever up to 39°C. An adenocarcinoma of the ileum had been diagnosed in June 2002 in this patient, and an ileocecal resection was performed followed by adjuvant chemotherapy. One year later, a local recurrence and peritoneal carcinomatosis were detected. The patient again underwent abdominal surgery by resection of ileo-colic anastomosis and sigmoid and peritoneal masses; a colostomy had to be created. The patient’s leukocyte count was 14.94 g/L (84.6% polymorphonuclear leukocytes), and his C-reactive protein level was 268 mg/L. Before antimicrobial drug therapy with amoxicilline/clavulanic acid and ciprofloxacin was begun, blood cultures were taken. One of the 2 anaerobic blood cultures was positive. Gram-negative bacilli were isolated (strain 4401054). Antimicrobial drug resistance was detected only to vancomycin. After receiving this therapy, the patient recovered rapidly.

Biochemical characterization was conducted by using API 20A and rapid ID 32A strips (bioMérieux, Marcy l’Etoile, France). Results were compared with those obtained for the reference strain *A. finegoldii* CIP 107999^T^. Strains 3302398 and 4401054 were indole positive and bile resistant, and they had positive enzyme reactions for N-acetyl-β-glucosaminidase, α-galactosidase, and β-galactosidase, as described for *A. finegoldii* (*4*). The 2 strains produced a brown pigment after 2 weeks’ incubation on sheep blood agar plates (bioMérieux).

PCR amplification of the 16S rDNA was performed with the primer pair fD1/rp2 (*5*). The generated fragments were sequenced as previously described (*6*). Sequences were compared with those available in GenBank databases by using BLAST (www.ncbi.nlm.nih.gov/blast). They showed a 97% identity to the 16S rDNA of *A. finegoldii* (accession nos. AY643083 and AY643084).

A novel bacterium was characterized from appendiceal tissues samples from children with appendicitis and in 2 cases of perirectal and brain abscesses associated with other anaerobes (*1*). With routine tests, this organism resembled members of the *Bacteroides fragilis* group; however, the cellular fatty acid composition dominated by *iso*-C15:0 and production of brown pigment on media containing hemolyzed blood suggested that the organism was most closely related to the genus *Porphyromonas* (*1*). However, 16S rDNA sequence comparison showed highest sequence relatedness with *B. putredinis,* and the reclassification of *B. putredinis* in a novel genus, *Alistipes*, and the classification of the novel bacterium as *A. finegoldii* were proposed (*4*). *A. putredinis* was characterized in the indigenous flora of the human gut (*7*). The natural habitat of *A. finegoldii* is unknown but is probably the same. *B. fragilis* is the most frequent anaerobic bacterium isolated from blood samples, and the principal source of the bacteria is the gastrointestinal tract (*8*). Predisposing factors to *Bacteroides* species bacteremia include malignant neoplasms, recent gastrointestinal or obstetric-gynecologic surgery, intestinal obstruction, and use of cytotoxic agents or corticosteroids (*8*). In both of our patients, fever was noted and no other microorganisms were isolated, indications that the bacteria probably were pathogenic.

Phenotypic identification of *Alistipes* sp. is difficult in a routine microbiology laboratory. However, a molecular approach based on 16S rRNA gene sequence comparison is a good method for identifying anaerobic bacteria, as it has recently been reported for *B. fragilis* in anaerobic sepsis (*9*) and for *B. thetaiotaomicron* from a patient with a cholesteatoma and purulent meningitis (*10*). In our 2 patients, we also used molecular identification because *A. finegoldii* was not included in the API phenotypic database identification. *A. finegoldii* should be considered as an agent of bacteremia in patients with gastrointestinal pathologic conditions.
